# Grafting of Polypyrrole-3-carboxylic Acid to the Surface of Hexamethylene Diisocyanate-Functionalized Graphene Oxide

**DOI:** 10.3390/nano9081095

**Published:** 2019-07-31

**Authors:** José Antonio Luceño-Sánchez, Ana Maria Díez-Pascual

**Affiliations:** Department of Analytical Chemistry, Physical Chemistry and Chemical Engineering, Faculty of Sciences, Alcalá University, 28805 Madrid, Spain

**Keywords:** polypyrrole, graphene oxide, hexamethylene diisocyanate, grafting reaction, morphology, thermal properties, tensile tests

## Abstract

A polypyrrole-carboxylic acid derivative (PPy-COOH) was covalently anchored on the surface of hexamethylene diisocyanate (HDI)-modified graphene oxide (GO) following two different esterification approaches: activation of the carboxylic acids of the polymer by carbodiimide, and conversion of the carboxylic groups to acyl chloride. Microscopic observations revealed a decrease in HDI-GO layer thickness for the sample prepared via the first strategy, and the heterogeneous nature of the grafted samples. Infrared and Raman spectroscopies corroborated the grafting success, demonstrating the emergence of a peak associated with the ester group. The yield of the grafting reactions (31% and 42%) was roughly calculated from thermogravimetric analysis, and it was higher for the sample synthesized via formation of the acyl chloride-functionalized PPy. The grafted samples showed higher thermal stability (~30 and 40 °C in the second decomposition stage) and sheet resistance than PPy-COOH. They also exhibited superior stiffness and strength both at 25 and 100 °C, and the reinforcing efficiency was approximately maintained at high temperatures. Improved mechanical performance was attained for the sample with higher grafting yield. The developed method is a valuable approach to covalently attach conductive polymers onto graphenic nanomaterials for application in flexible electronics, fuel cells, solar cells, and supercapacitors.

## 1. Introduction

Polypyrrole (PPy) and its derivatives are conductive polymers that have been extensively investigated over recent years due to their potential applications in a variety of fields including organic solar cells (OSCs), organic field-effect transistors (OFETs), organic light-emitting diodes (OLEDs), rechargeable batteries, supercapacitors, antistatic coatings, textile coatings, transducers in chemical sensors and biosensors, actuator components and fuel cells, among others [1,2,3]. Moreover, the application of these conducting polymers as biodegradable materials for biomedical uses (e.g., neurological implants, drug delivery, cell and tissue engineering) is currently being explored [4]. PPy is electrically insulating, and conductivity is achieved by doping, either by partial oxidation (p-doping) or reduction (n-doping) in an electrochemical cell or via redox reagents [5]. The mechanism of charge transport can be understood considering the formation of polarons (radical cations) at low oxidation levels and bipolarons at high oxidation levels [6]. Further, electron hopping can take place between polymer chains.

PPy can be obtained by polymerization in aqueous solution, and shows good electrical conductivity, environmental stability against water and air, facile synthesis via both electrochemical and chemical strategies, and capability of copolymerization without sacrificing electroactive performance [2]. However, PPy has low solubility in common organic solvents, which restricts its processability, as well as low thermal and electrical stability, poor mechanical properties, reduced charge/discharge rate capability and a limited cycle stability, which limit its practical applications. A common strategy to overcome these shortcomings is the formation of composites with other materials such as metal oxides or carbon nanomaterials [7]. Accordingly, carbon nanotubes (CNTs), graphene (G) and its derivatives have been used as fillers to spread the practical uses and enhance the performance of conductive PPy. For instance, the incorporation of CNTs enables a faster charge/discharge process due to enhanced conductivity, and provides mechanical reinforcement that accommodates the associated volumetric changes [8]. PPy/G nanocomposites have been prepared via in situ chemical or electrochemical polymerization, leading to better conductivity and specific capacitance and electrochemical behavior [7,9,10]. However, the improvements attained are generally insufficient to fulfill the requirements of the commercial applications. Owing to their large surface area, G nanosheets have a strong tendency to form irreversible agglomerates or even restack due to van der Waals interactions, which limits property improvements [11], and such issues must be resolved to obtain a satisfactory performance of the final G-based nanocomposites. In this regard, novel strategies are pursued to improve the G dispersion and its interfacial adhesion with the PPy matrix. 

Currently, the mixture of G and its derivatives with polymers is a very challenging task; to attain stable G dispersions and a good control of the nanocomposite microstructure, non-covalent or covalent functionalization approaches are generally necessary [12]. The non-covalent functionalization, which is based on van der Waals forces, electrostatic interactions or π-π stacking [13], is simpler to perform without modifying the structure of the G nanosheets, and offers efficient ways to fit the electronic/optical properties and dispersion ability of the nanosheets [14]. The covalent functionalization is typically carried out via a chemical reaction between the surface oxygen groups of graphene oxide (GO) or reduced graphene oxide (rGO) [12], namely, epoxide and hydroxyl located on the basal planes and carboxylic acids on the edges with functional groups of molecules or polymers. Both “grafting to” and “grafting from” approaches have been employed to covalently anchor polymeric segments onto the GO surface. The “grafting from” method consists of first grafting the monomers on the GO surface, followed by in situ polymerization [12]. Nonetheless, this procedure might not be feasible in some cases, in which the covalent connection between the presynthesized polymer and GO arises as the solely option. To extend the number of polymers that can be linked to GO, the “grafting to” approach can be employed, which involves the union of functionalized end polymer chains to the GO surface. Compared with the non-covalent approach, the covalent functionalization of G nanosheets shows higher versatility and potential due to the abundant surface chemistry of GO/rGO [15]. Nonetheless, it is important to note that although the covalent attachment of GO or rGO to polymeric chains improves some properties, it may have a detrimental effect on others, especially those associated with the motion of electrons or phonons [16]. Therefore, control of the degree of grafting and the GO/polymer weight ratio in the composites is essential in order to attain an optimum balance of properties [17]. 

Polymers incorporating functional groups such as amino, hydroxyl, or carboxylic acid can be covalently linked to GO. For example, the nucleophilic ring-opening reaction between the epoxy groups of GO and amine groups has been broadly used [18]; other strategies are the esterification/amidation reactions between the carboxyl groups of GO and the hydroxyl or amine groups of polymers [12]. Conductive polymer-functionalized graphene materials have also been prepared by esterification/amidation processes [19]. As a result, the solubility of the synthesized materials in common solvents has been considerably improved, enabling device preparation by solution processing. Thus, composites obtained by chemical grafting of poly(3-hexylthiophene) (P3HT) chains onto GO have been incorporated into specific devices by simple spin coating, exhibiting higher power conversion efficiency for solar cell applications [20]. Further, by using this covalent binding reaction, a 3D cross-linked network was formed that increased the stiffness and strength of the composites.

In a preceding work [21], a series of hexamethylene diisocyanate (HDI)-modified GO derivatives with different functionalization degrees (FD) were synthesized by a simple reaction between organic HDI and GO using triethylamine (TEA) as a catalyst. The derivatives with higher FD showed better thermal stability than pristine GO and a more hydrophobic character; hence, they were suitable candidates to be incorporated as fillers into conductive polymers. In the present study, a polypyrrole-carboxylic acid derivative (PPy-COOH) was synthesized via chemical polymerization, which was covalently grafted to the surface of the HDI-GO with the highest FD. The grafting of the PPy-COOH chains to HDI-GO was performed via two different esterification reactions: the first was carried out via activation of the carboxylic acids of the polymer by carbodiimide, whereas the second was via reaction with thionyl chloride to yield the acyl-chloride-modified PPy. The resulting PPy-COOH-*g*-HDI-GO samples have been examined and compared with the characteristics of PPy-COOH, in order to get insight about the yield of the grafting processes as well as their morphology, structure, thermal, electrical, and mechanical properties.

## 2. Materials and Methods

### 2.1. Reagents

Pyrrole-3-carboxylic acid (C_5_H_5_NO_2_ > 97%, M_w_ = 111.1 g/mol, d_25°C_ = 0.862 g/cm^3^), ammonium persulfate ((NH_4_)_2_S_2_O_8_, 98%, M_w_ = 228.20 g/mol, d_25°C_ = 1.98 g/cm^3^), dimethylformamide (DMF, 99% C_3_H_7_NO, M_w_ = 73.09 g/mol, d_25°C_ = 0.944 g/cm^3^), N,N-dicyclohexylcarbodiimide (DCC, 99%, C_13_H_22_N_2_, M_w_ = 206.33 g/mol; d_25°C_ = 1.32 g/cm^3^), 4-dimethylaminopyrydine (DMAP, 99%, C_7_H_10_N_2_, M_w_ = 122.17 g/mol, d_25°C_ = 0.96 g/cm^3^), and thionyl chloride (SOCl_2_, 97%, M_w_ = 118.96 g/mol, d_25°C_ = 1.64 g/cm^3^) were obtained from Sigma-Aldrich (Madrid, Spain). Hexamethylene diisocyanate-modified graphene oxide (HDI-GO), with a functionalization degree of 18.1%, was synthesized according to our previous works [22,23]. The organic solvents (HPLC grade) were obtained from Scharlau S.L. (Barcelona, Spain). High purity deionized water was obtained from a Millipore water purification system (≥18 MΩ). The monomer and DMF were distilled under vacuum prior to use; the rest of the reagents were used as received.

### 2.2. Synthesis of poly(pyrrole 3-carboxylic Acid) (PPy-COOH)

The polypyrrole derivative was prepared by chemical polymerization in acid medium using (NH_4_)_2_S_2_O_8_ as oxidizing agent. 1 M pyrrole-3-carboxylic acid solution was prepared using distilled water, and polymerization was initiated by adding (NH_4_)_2_S_2_O_8_ in HCl solution dropwise (monomer/oxidant ratio of 1:2.4). The mixture was then stirred while cooling in an ice bath for 24 h. The resulting precipitate PPy-COOH was filtered and washed repeatedly with deionized water and acetone to eliminate the unreacted PPy and the short oligomer chains until the filtrate was clear and colorless, then dried under vacuum for 12 h at 70 °C. 

### 2.3. Grafting of PPy-COOH to HDI-Modified GO 

**Scheme 1:**
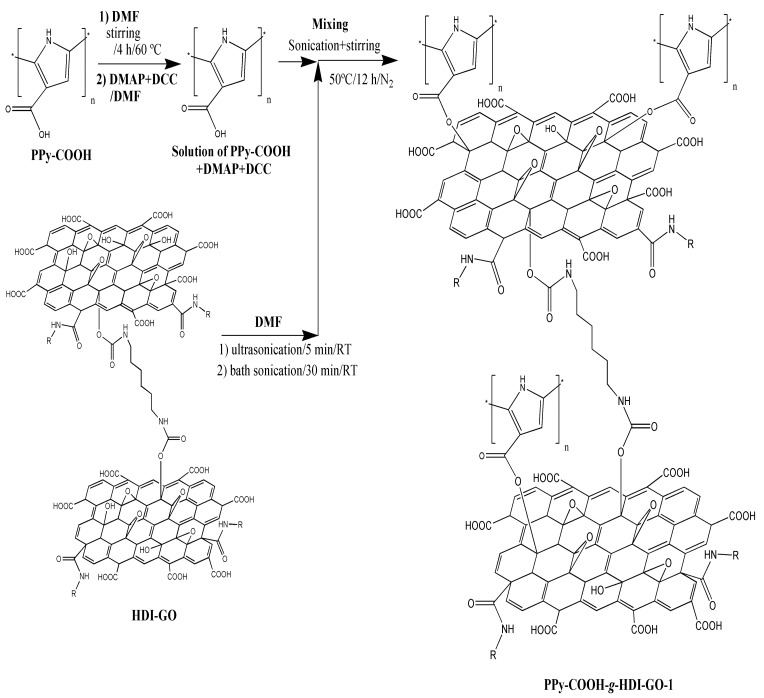
Schematic representation of the synthesis of PPy-COOH-g-HDI-GO-1 via activation of the carboxylic acids of the polymer by carbodiimide.

The grafting of the polypyrrole derivative to the HDI-GO was performed following two different esterification reactions. In the first one, the carboxylic acids of the polymer were activated by carbodiimide (Scheme 1). HDI-GO (30 mg) was dispersed in DMF (10 mL) by point-probe sonication (5 min, 40% amplitude) followed by bath sonication for 30 min.

Separately, PPy-COOH (300 mg) was dispersed in DMF (10 mL) and kept at 60 °C for 4 h under moderate stirring. Then, the PPy-COOH dispersion was mixed with a solution of DCC (0.825 g, 4 mmol) and DMAP (0.06 g, 1.0 mmol) in DMF (20 mL). Subsequently, both PPy-COOH and HDI-GO dispersions were mixed via sonication and mild stirring, and the reaction was allowed to proceed at 50 °C for 12 h under a nitrogen atmosphere. The resulting grafted product, hereafter designated as PPy-COOH-*g*-HDI-GO-1, was filtered, washed repeatedly with methanol, and vacuum dried at 50 °C for 24 h.

**Scheme 2:**
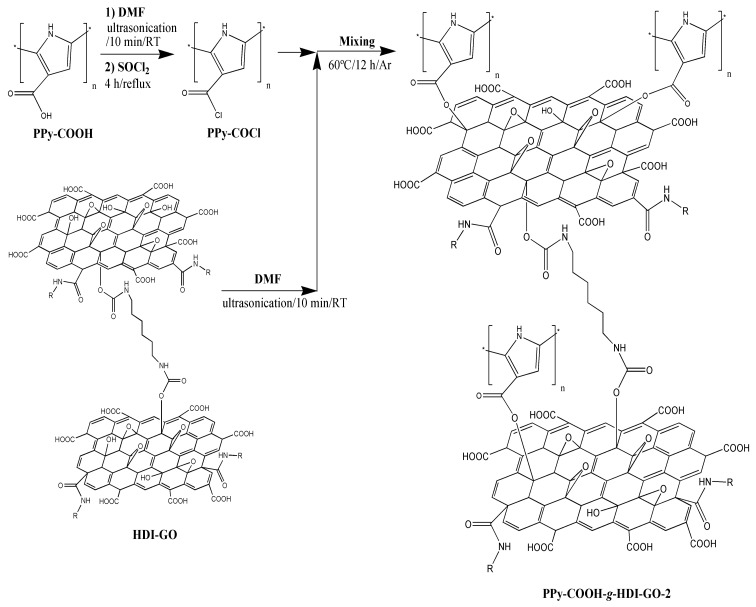
Schematic representation of the synthesis of PPy-COOH-*g*-HDI-GO-2 via reaction of the carboxylic groups with SOCl_2_.

The second approach was performed via reaction of the carboxylic groups of the polymer with thionyl chloride to yield the acyl chloride derivative (Scheme 2). Firstly, PPy-COOH (300 mg) was dispersed in DMF (10 mL) and sonicated in a bath for 10 min. Then, an excess of SOCl_2_ (1 mL, 13.7 mmol) was added and the reaction took place for 4 h under reflux and constant stirring. Subsequently, the residual SOCl_2_ was eliminated by distillation under vacuum to produce the acyl-chloride-modified polymer (PPy-CO-Cl). Separately, HDI-GO (30 mg) was dispersed in DMF (10 mL) by sonication with the ultrasonic probe for 5 min combined with bath sonication for 30 min. Then, pyridine (2 mL) was added and the mixture was kept under stirring at 60 °C. After, PPy-CO-Cl was mixed with the HDI-GO dispersion and the reaction continued for 12 h at 60 °C under stirring and an inert atmosphere. The resulting compound, hereafter named as PPy-COOH-*g*-HDI-GO-2, was filtered, washed repeatedly with methanol, and dried under vacuum at 50 °C.

### 2.4. Instrumentation

Samples were weighed with a Sartorious Cubis Ultramicro Balance (Leicestershire, UK), readability ±0.0001 mg. An ultrasound bath (200 W, Ultrasons, Selecta) and a Hielscher UP400S ultrasonic tip (24 kHz, 400 W, Teltow, Germany) set with a titanium sonotrode of 7 mm diameter and 100 mm length were used to prepare the PPy-COOH and HDI-GO dispersions.

The surface morphology of the samples was examined with a Hitachi SU8000 cold field emission scanning electron microscope (SEM, Hitachi, Ltd., Tokyo, Japan) at an acceleration voltage of 15.0 kV and an emission current of 10 mA. Before the experiments, samples were covered with a 5-nm thick Au:Pd overlayer to prevent charge accumulation throughout electron irradiation. The dispersions were also examined by transmission electron microscopy (TEM) with a Zeiss EM-10C/CR instrument (Oberkochen, Germany) operating at a voltage of 60 kV.

Fourier transform (FT-IR) spectra were recorded at 25 °C in transmission mode between 600 and 4000 cm^−1^, at a resolution of 4 cm^−1^ and an incident laser power of 1 mW, using a Perkin-Elmer Spectrum One spectrometer (PerkinElmer Inc., Massachusetts, MA, USA) set with an ATR sampling accessory (diamond crystal) and a laser excitation source (632.8 nm). Typically 10 scans were collected for each sample to improve the signal-to-noise ratio.

Raman spectra were recorded at 25 °C in confocal mode, at a laser power of 1 mW, with a Renishaw InVia Raman spectrometer (Gloucestershire, UK), equipped with a Peltier-cooled CCD detector, a Leica microscope, and a He-Ne laser (632.8 nm). Spectra were analyzed with the WiRE 3.3 Renishaw software. 50 scans were acquired for each sample to reduce the signal-to-noise ratio. Thermal characterization of the samples was carried out by thermogravimetric analysis (TGA) using a TA Instruments Q500 thermobalance (Barcelona, Spain) coupled with a mass spectrometer under a nitrogen atmosphere. After drying for 72 h, sample masses ranging from 5 to 10 mg were put in aluminum oxide crucibles, then heated from 100 up to 600 °C at a heating rate of 10 °C/min under a purge gas flow rate of 60 mL/min. 

X-ray diffraction (XRD) patterns were recorded with a Bruker D8 Advance diffractometer (Karlsruhe, Germany) using CuKα (λ = 1.54 Å), with a voltage of 40 kV and an intensity of 40 mA.

Brunauer–Emmett–Teller (BET) specific surface area (total surface area per unit of mass) was calculated from N_2_ adsorption with a Micromeritics ASAP 2020 Plus analyzer (Norcross, GA, USA) at liquid nitrogen temperature. 

The sheet resistance (R_S_) was determined using a four-point probe (Multiheight Probe station, Leighton Buzzard, UK) connected to a nanovoltmeter (Keithley 2182a, Bracknell, UK) and a current source (Keithley 6221). The sheet resistance was calculated as: Rs = 4.532(U/I), where U is the measured voltage (V) between the inner probes, and I is the current (A) through the outer probes. To ensure reproducibility, more than five measurements at different points of each sample were carried out.

Tensile curves were obtained with an Instron 5565 Tester (Norwood, MA, USA), applying a 1-kN load cell at 50% relative humidity (RH) and a crosshead speed of 10 mm/min, both at 25 and 100 °C. Data reported correspond to the average value of at least six replicates.

## 3. Results

### 3.1. Morphology of PPy-COOH-g-HDI-GO Grafted Samples

**Figure 1:**
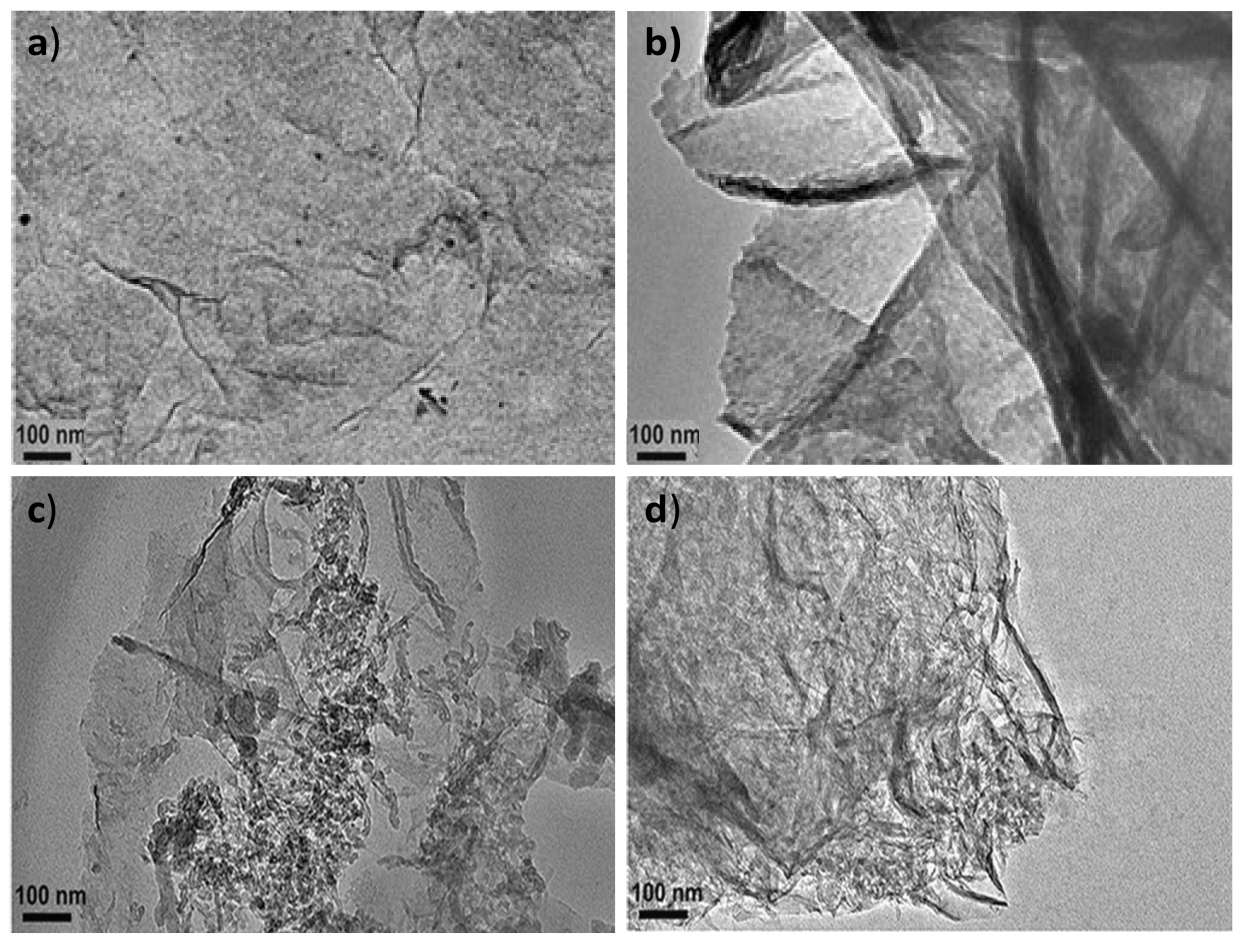
Representative TEM micrographs of PPy-COOH (**a**), HDI-GO (**b**), PPy-COOH-*g*-HDI-GO-1 (**c**), and PPy-COOH-*g*-HDI-GO-2 (**d**).

The surface morphology of PPy-COOH, HDI-GO, and the grafted nanocomposites was assessed by TEM, and typical images are compared in Figure 1. PPy-COOH (Figure 1a) shows a homogenous somewhat-coarse surface, with good film-forming ability. Nonetheless, its morphology is difficult to be visualized by TEM, due to its amorphous character and the poor contrast of the polymeric functional groups.

On the other hand, the HDI-GO samples display a homogeneous and uniform appearance (Figure 1b), with a stacked-like structure, comprising flexible and slightly curled flakes with an average thickness of 20 nm. It should be noted that this thickness is significantly higher than that reported for a single monolayer graphene (about 0.34 nm [24]) due to the presence of oxygen-containing functional groups and the alkyl chains of the organic diisocyanate attached on both sides of the flakes. Further, each sample contains several modified graphene layers. The darker areas in Figure 1b are probably related to different thicknesses or disposition of the nanosheets due to the presence of the HDI. This is in agreement with former observations described by other authors who anchored alkyl or polymeric chains onto graphene [25,26]. In accordance with those works, the grafted chains extend into the solution, but in the solid state the chains collapse onto the GO surface, forming small domains that probably correspond to more-obscure features. It is also important to mention that the HDI-GO samples show a lower degree of folding than typically observed for pristine GO [27], since the alkyl chains can wrap around the GO nanosheets and shield the wrinkles.

Regarding the PPy-COOH-g-HDI-GO-1 sample (Figure 1c), it appears as a heterogeneous mixture with continuous clearer areas attributed to the polymer and darker regions corresponding to the HDI-GO. It is important to note that the grafting reactions are not quantitative, due to steric effects, and hence the grafted samples are composed of regions in which the nanofiller is covalently bonded to the monomer matrix material, as well as parts where the nanofiller interacts with the monomers via non-covalent interactions, as will be discussed later. Interestingly, the average flake thickness is slightly decreased compared to HDI-GO, likely because the polymeric chains intercalated between the nanosheets and induced exfoliation. This is in contrast to some previous studies on polymer grafted-graphene samples, in which the thickness increased upon the covalent bonding to the polymeric segments [28]. This discrepancy can be explained considering two phenomena that can take place upon the PPy-COOH grafting: on the one hand, the presence of polymer chains onto the nanofiller surface can increase layer thickness, while on the other hand the intercalation of the chains within the GO nanosheets can cause exfoliation, thus reducing layer thickness. It seems that in this case the intercalation process predominates, resulting in a lower flake thickness overall.

The PPy-COOH-g-HDI-GO-2 (Figure 1d) appears more homogeneous compared to the other grafted sample, and the darker areas corresponding to the HDI-GO are less visible. It presents a rougher and more wrinkled surface, suggesting the presence of the PPy-COOH wrapping around the GO nanosheets, forming a tight interfacial layer, without voids or discontinuities, indicating good interfacial adhesion and compatibility between the two nanocomposite phases. The average flake thickness is slightly higher than that found for the HDI-GO, suggesting that, in this case, the grafting of the polymer chains onto the GO surface prevails. All these observations confirm that the PPy-COOH is physically and/or chemically bonded to the surface of the HDI-GO.

BET specific surface area (SSA) was measured to get further insight about the influence of the esterification reactions on the level of exfoliation of the GO nanosheets in the different samples, and the results obtained are collected in Table S1 in the Supplementary Materials. A high SSA is interesting for a variety of applications such as supercapacitors [29]. It is known that the SSA of graphene-based nanomaterials increases as the sheet thickness decreases [15]. The SSA of GO (about 100 m^2^/g) slightly decreased upon functionalization with HDI, which is reasonable considering that the incorporation of the alkyl chains reduces the sp^2^ surface area. On the other hand, the SSA of PPy-COOH is close to 25 m^2^/g, and increased upon HDI-GO grafting, in agreement with previous studies on PPy/graphene nanocomposites [29]. This increment was more significant for PPy-COOH-g-HDI-GO-1, in agreement with its thinner flakes and better level of exfoliation, as revealed by TEM. 

**Figure 2:**
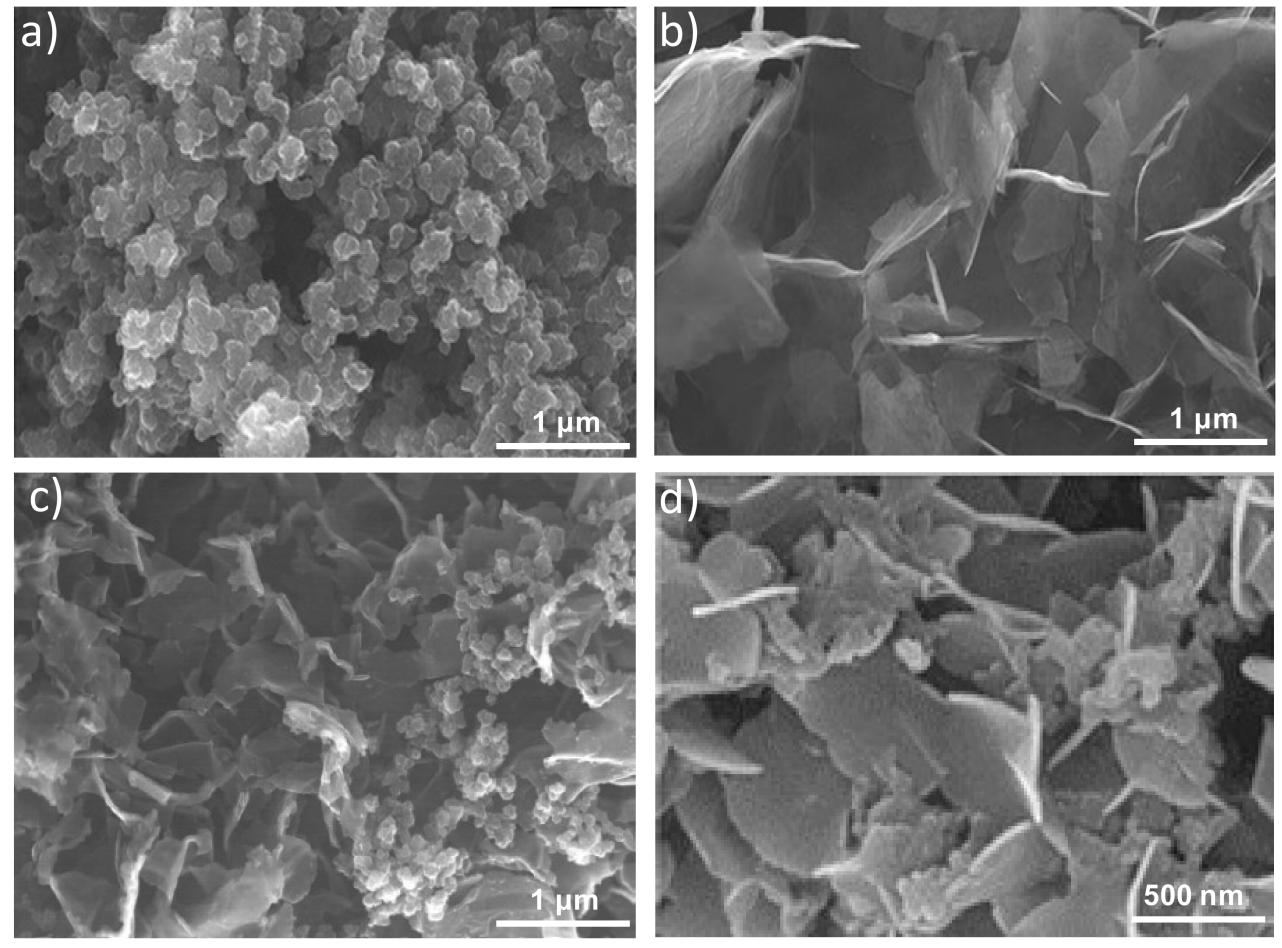
SEM images of PPy-COOH (**a**), HDI-GO (**b**), and PPy-COOH-*g*-HDI-GO-2 (**c**). The image in (**d**) is a magnification of (**c**) showing the HDI-GO nanosheets dispersed within the PPy-COOH.

Further information about the morphology of the samples was attained by SEM, and typical images are shown in Figure 2. Figure 2a reveals nano- and meso-particles of PPy-COOH with spherical morphology and a smooth surface arranged in random clusters showing an average particle diameter of 110 nm. Regarding the HDI-GO (Figure 2b), it is composed of flexible and quite well-exfoliated flakes, with thicknesses between 10 and 50 nm and lateral sizes of 1–2 μm. Conversely, PPy-COOH-*g*-HDI-GO-2 (Figure 2c) appears as a homogenous sample with the graphene nanosheets randomly dispersed within the PPy-COOH particles. The sheets are less bendable and appear with a smoother surface than those in HDI-GO, probably due to the grafting of the polymeric, and appear thicker, with thicknesses of up to 70 nm, as can be observed at a higher magnification (Figure 2d). Similar morphology, albeit with thinner sheets and uneven HDI-GO dispersion, was found for PPy-COOH-*g*-HDI-GO-1. From all the above observations, it can be inferred that the esterification reaction via acylation with thionyl chloride leads to higher grafting yields, hence larger amounts of polymer chains anchored to the nanofiller surface, which will be discussed in the following sections.

### 3.2. IR and Raman Spectroscopies

**Figure 3:**
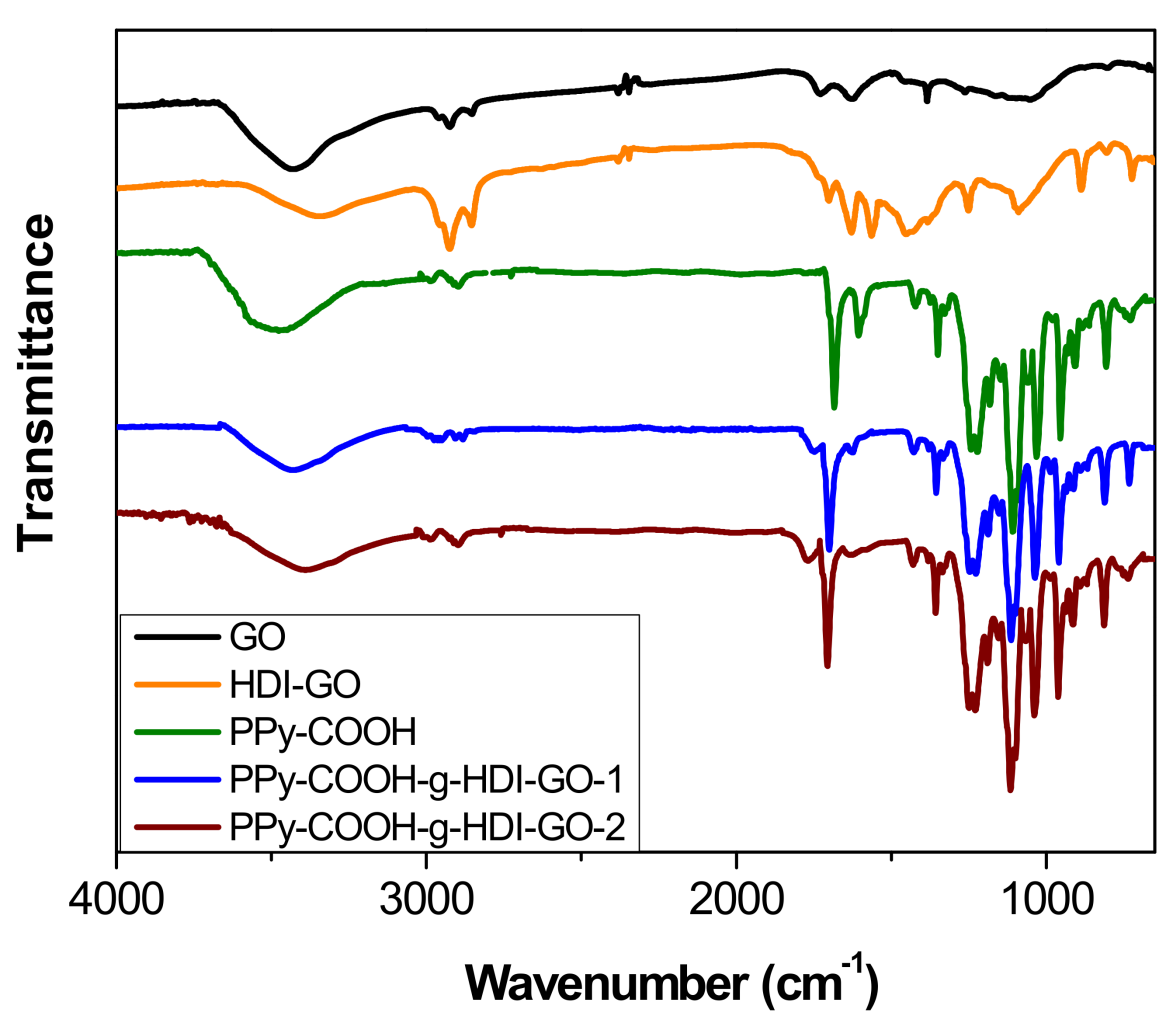
FT-IR spectra of neat GO, HDI-GO, PPy-COOH, and the two grafted samples.

The chemical changes that took place after treatment of PPy-COOH with HDI-GO were examined by FT-IR spectroscopy (Figure 3). For comparative purposes, the spectrum of pristine GO was also examined. Neat GO encloses epoxide and hydroxyl groups on the basal planes and carboxylic acids at the flake edges [11,15]. The most distinctive characteristics in the IR spectrum of GO are found at ~3435 cm^−1^, ascribed to the O–H stretching; at about 2930 and 2850 cm^−1^, attributed to sp^2^ and sp^3^ C–H stretching vibrations (formed at defect sites of the aromatic system); at 1730 cm^−1^ due to the C=O stretching of the carboxylic group; at ~1620 cm^−1^, ascribed to the C–C stretching of the aromatic network; at around 1400 cm^−1^, corresponding to the O–H deformation; and at 1260 cm^−1^, due to the C–OH stretching [30].

Upon treatment with the HDI, the strength of the O–H stretching decreased and the band shifted to lower wavenumbers, due to the formation of H bonds between the residual OH moieties and other oxygenated groups close to them. Further, the shift should also be a result of the overlapping with the N–H stretching of the carbamate groups. Nonetheless, it should be taken into account that the intensity of the O–H stretching peak can also be influenced by the presence of environmental humidity, and hence cannot be used as a conclusive proof of HDI grafting. Moreover, the peaks at ~2930 and 2850 cm^−1^ originated from asymmetrical and symmetrical stretching vibrations of the methylene chains of the HDI, respectively. Further, instead of the peak at 1730 cm^−1^ corresponding to the C=O stretching of carboxylic acids, a new band appears at ~1710 cm^−1^, ascribed to the C=O of the carbamate esters [21]. In addition, strong novel bands arising from the carbamate ester groups can be observed at about 1650 and 1580 cm^−1^, the first due to the coupling of the C=O stretching with the N–H bending, and the second due to the coupling of the N–H bending with the C–N stretching [31]. Other peaks can also be found at about 1110 cm^−1^, related to –C(=O)–O and C–N stretching vibrations of the carbamate groups; at 880 cm^−1^, attributed to C–H out-of-plane bending vibrations of the substituted aromatic rings; and at around 720 cm^−1^, due to the rocking of the methylene groups of HDI.

Regarding the PPy-COOH, an intense and broad band can be found centered at 3482 cm^−1^, due to the overlapping of O–H and N–H stretching vibrations. The peak at 1673 cm^−1^ corresponds to the C=O stretching of the carboxylic acid, the peaks at 1580 and 1430 cm^−1^ are assigned to fundamental vibrations of the pyrrole rings, and those at about 1200 and 920 cm^−1^ correspond to the bipolaron bands [32], indicating that the synthesized PPy-COOH is in an oxidized state.

**Scheme 3:**
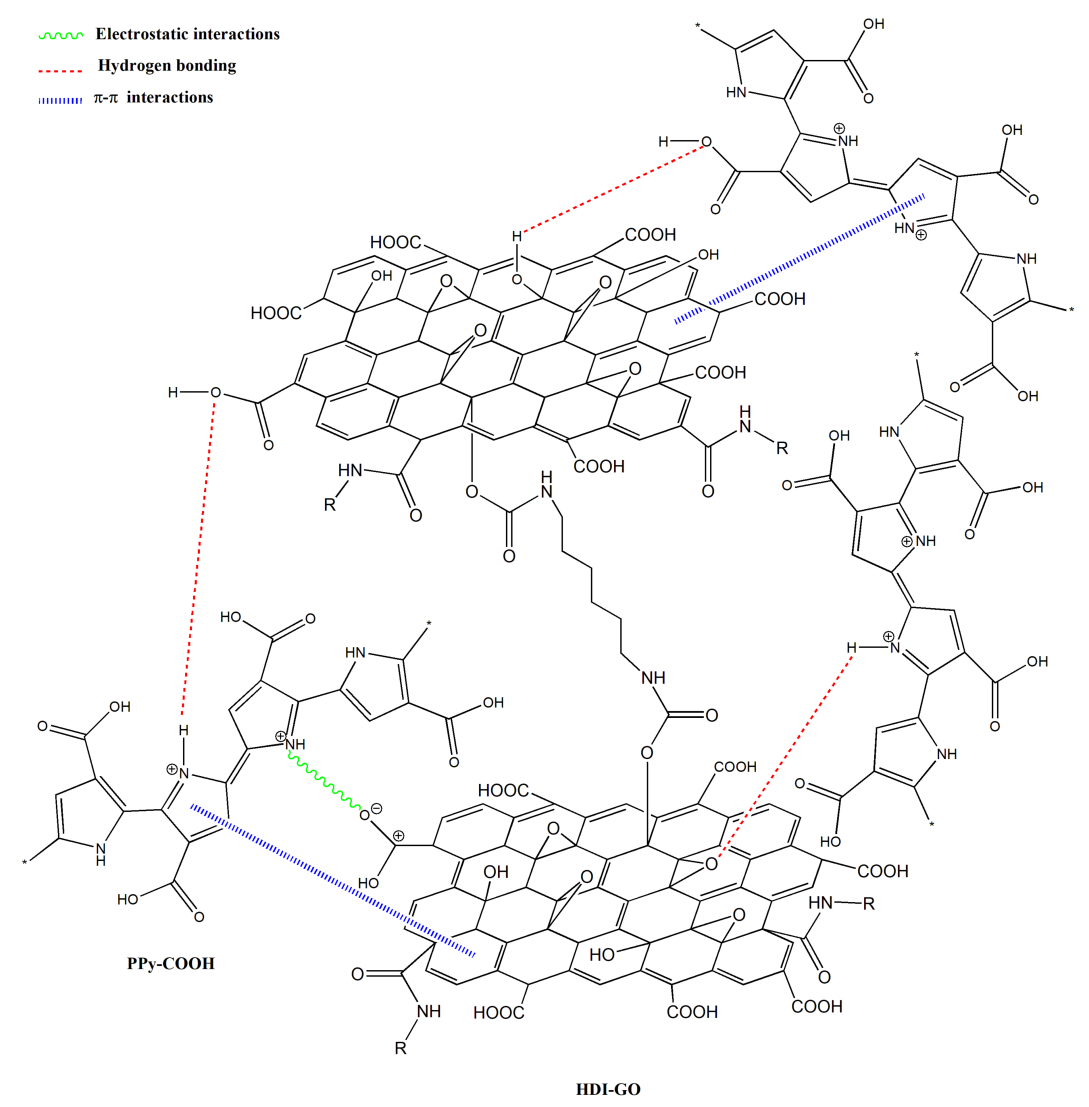
Representation of the non-covalent interactions between the bipolaron structure of PPy-COOH and HDI-GO.

The peaks of HDI-GO cannot be observed in the spectra of PPy-COOH-g-HDI-GO-1 or PPy-COOH-g-HDI-GO-2, which is reasonable considering that the total amount of this modified GO is only 9% of the weight of the grafted samples, hence its bands should be very weak and masked by the polymer ones. Thus, the spectra of both samples is quite similar to that of the polymer, albeit with decreased intensity of the O–H stretching band due to the esterification of part the hydroxyl groups with the carboxylic acids of PPy-COOH. Nonetheless, as mentioned earlier, this reduction should be considered only as indicative since the peak intensity could have been affected by the stretching vibration of the water molecules in the environment. Further, the band is significantly shifted to lower wavenumbers, and appears at 3430 and 3388 cm^−1^ for PPy-COOH-g-HDI-GO-1 and PPy-COOH-g-HDI-GO-2, respectively. Besides, the band corresponding to the carboxylic acid shifts up to 1699 and 1708 cm^−1^, respectively, and this behavior might be an indication of H-bonding interactions between the OH and NH moieties of PPy-COOH and the oxygenated functional groups of the GO surface [33,34] (see Scheme 3). In addition, strong π-π stacking interactions take place between the aromatic rings of pyrrole and those of GO, as well as electrostatic interactions between the negatively charged carboxylic acid groups of the HDI-GO surface and the positively charged PPy-GOOH rings in the oxidized state (bipolaron structure), which would also induce a shift in the band positions. More importantly, after the grafting reactions, a new band appears at 1743 and 1760 cm^−1^ for PPy-COOH-g-HDI-GO-1 and PPy-COOH-g-HDI-GO-2, respectively, related to the C=O stretching of the ester group. The appearance of this band in the spectra of both grafted samples is one of the outmost outcomes derived from the IR analysis, given that it corroborates that part of the carboxylic acids of PPy-COOH have reacted with the hydroxyl moieties of the HDI-GO. 

**Figure 4:**
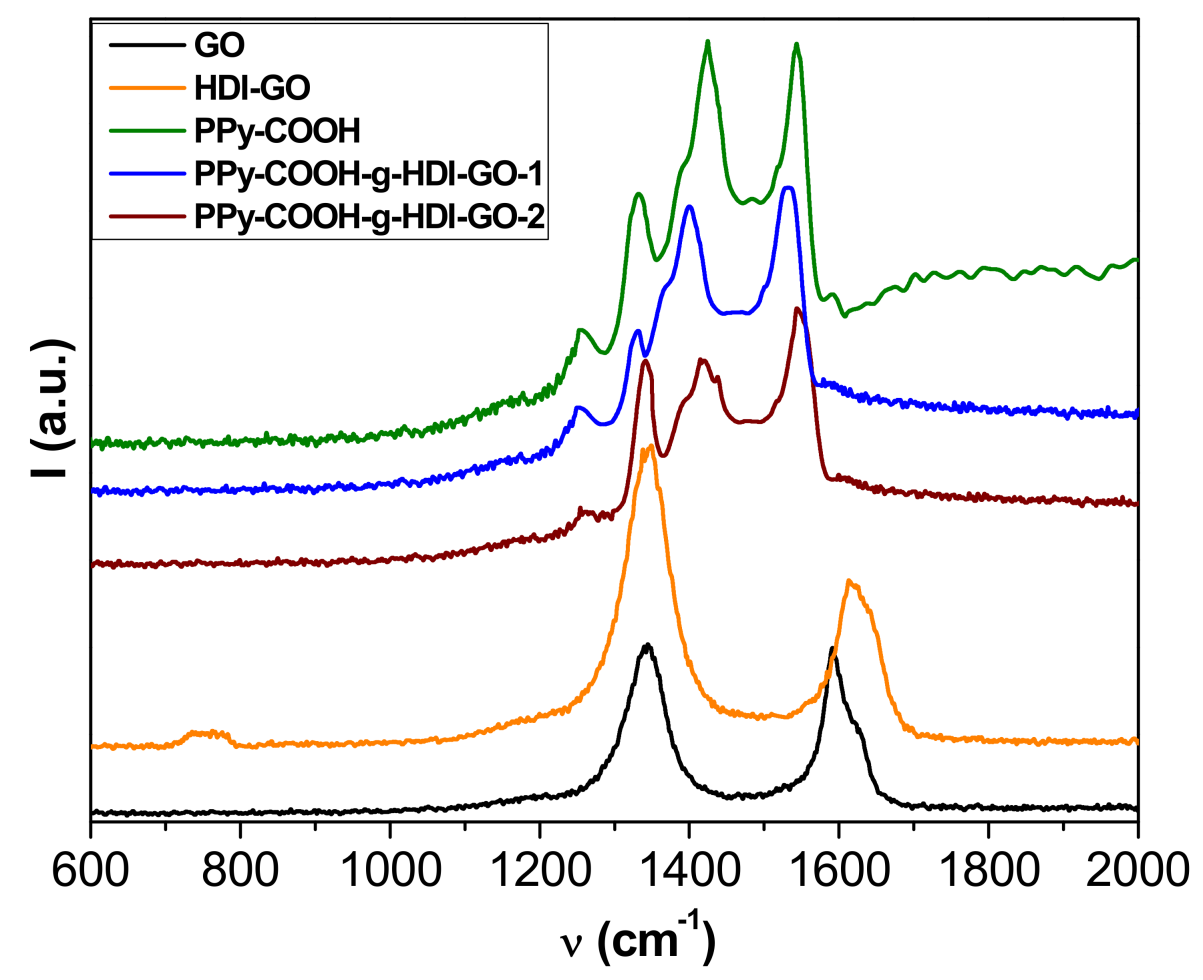
Raman spectra of GO, HDI-GO, PPy-COOH, PPy-COOH-*g*-HDI-GO-1, and PPy-COOH-*g*-HDI-GO-2.

More information about the interactions between the polymer and the nanofillers was obtained from Raman Spectroscopy, and the spectra obtained from the different samples are depicted in Figure 4. The Raman spectrum of GO shows two prominent bands: the defect D band that accounts for the structural disorder at 1354 cm^−1^, and the tangential G band arising from graphitized structures at about 1600 cm^−1^ [29]. An analogous spectrum is found for HDI-GO, although it shows a widening and an upshift of the G-band, attributed to a modification in the electronic structure of GO when electron-acceptor groups are incorporated into the nanosheets [21]. Moreover, this upshift can be linked to a rise in the concentration of defects in the graphene sheets. It is important to note that the D to G band intensity ratio (*I*_D_/*I*_G_) gives quantitative information on the concentration of defects in graphene flakes—the higher the ratio, the larger the amount of defects [22]. This ratio increases by 1.74 from GO to HDI-GO, which demonstrates a fall in the structural order after anchoring the GO to the HDI chains. 

The spectrum of PPy-COOH exhibits five main features [35]: the C–C inter-ring stretching at 1260 cm^−1^, the single C–C stretching at 1330 cm^−1^, the C-N stretching at 1390 cm^−1^, the C=C symmetrical stretching at 1430 cm^−1^, and the C=C antisymmetrical stretching at 1550 cm^−1^. Regarding the spectra of the grafted samples, the D and G bands of HDI-GO cannot be observed because they are likely very weak and masked by the polymer bands. On the other hand, a drop in the intensity of the bands of the polymer and a shift in their position is detected. For the PPy-COOH-g-HDI-GO-1, the bands are shifted towards lower wavenumbers, which could be due to the rupture and deformation of bonds induced by the grafting process [36]. In contrast, the bands in the PPy-COOH-g-HDI-GO-2 are slightly upshifted, likely because the grafting of polymeric chains onto the HDI-GO surface prevails (as revealed by TEM images), together with the adsorption of the PPy-COOH segments onto the nanofiller via π-π stacking and H-bonding interactions, which can lead to the formation of a tightly coated polymeric layer on the nanomaterial surface. These shifts in the peaks confirm again the intense interactions between the polymer derivative and the modified GO. Analogous behavior of the shift of the Raman bands has previously been reported for poly(N-vinyl carbazole)/PPy/rGO composites [37]. 

### 3.3. Thermal Stability and Yield of the Grafting Reaction

**Figure 5:**
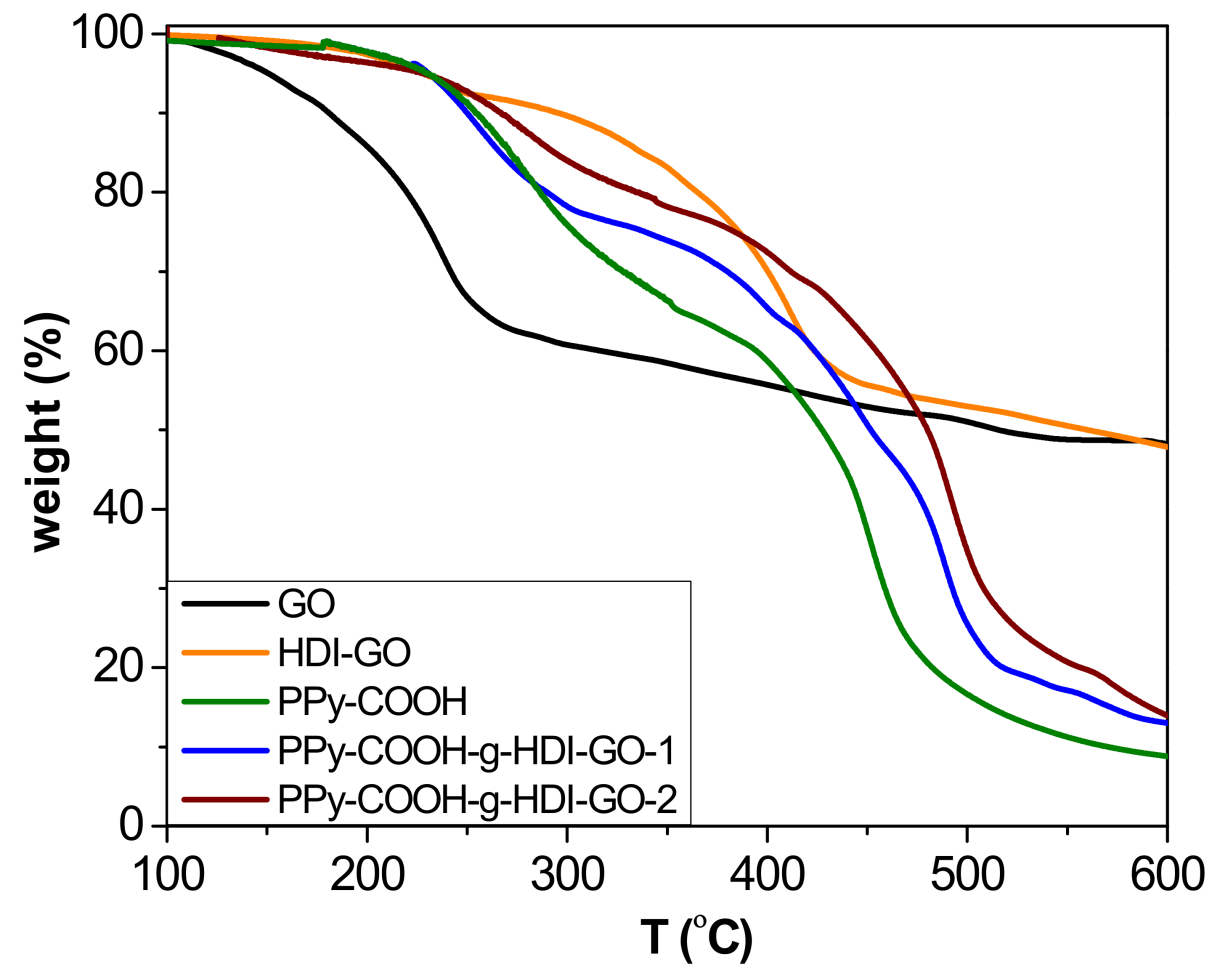
TGA curves under inert atmosphere of GO, HDI-GO, PPy-COOH, and the grafted samples.

TGA analysis under a nitrogen atmosphere was carried out to investigate the thermal stability of the synthesized PPy-COOH and the grafted samples (Figure 5), and to obtain information about the yield of the grafting reactions. The esterification reactions carried out in this work do not proceed in quantitative yields due to steric effects, and the efficiency depends on the esterification route. The thermobalance was coupled to a mass spectrometer, enabling identification of the degradation products. All the results derived from TGA analysis are collected in Table S1 (Supplementary Materials). Pristine GO shows a one-step degradation process, with the most important weight loss at temperatures below 240 °C attributed to the removal of epoxide, hydroxyl, and carboxylic acid surface groups [21]. In addition, a small weight loss is observed at higher temperatures due to the elimination of more functional groups. In contrast, the HDI-GO presents two decomposition steps, the former arising from the removal of remaining surface oxygen-containing groups, and the other to the degradation of the HDI moieties connected to the GO surface. The incorporation of the alkyl chains onto the nanomaterial surface results in a significant improvement in thermal stability, leading to an increase in the initial degradation temperature (T_i_) of about 70 °C. On the other hand, the PPy-COOH exhibits a weight loss in the range of 200–380 °C that is associated with a fragment of m/z 45 (corresponding to elimination of the carboxylic acids [38]), as well as a second weight loss between 380 and 500 °C related to the decomposition of the polymeric backbone, which exhibits the maximum rate (T_max_) at 444 °C. Regarding the grafted samples, two major weight losses can also be observed that are similar to the parent polymer, the first taking place in the same temperature range while the second is shifted to higher temperatures. Taking into account the weight loss of the first stage, the amount of COOH groups chemically bound to the OH moieties of the HDI could be predicted (considering the experimental error). The difference between the weight loss of this step for the polymer derivative and the grafted samples obtained after the esterification process corresponds to the yield of the grafting reaction [16]: ~31% and 42% for PPy-COOH-g-HDI-GO-1 and PPy-COOH-g-HDI-GO-2, respectively. Thus, it is confirmed that the esterification approach via reaction of the carboxylic groups with SOCl_2_ was more effective, leading to higher reaction yields. 

The second step is associated with the decomposition of the PPy-COOH backbone, which occurs at higher temperatures in the grafted samples than the PPy-COOH. Thus, for PPy-COOH-g-HDI-GO-1 and PPy-COOH-g-HDI-GO-2, T_max_ of this second stage occurs about 30 and 40 °C above that of the neat polymer, respectively. The higher thermal stability of the second sample is ascribed to its higher level of grafting. This enhancement in the maximum degradation rate is associated with the HDI-GO alkyl chains covalently bonded to the polymeric segments, which successfully hinder the transport of the degradation products from the interior of the matrix to the gas phase. In addition, the higher decomposition temperatures indicate that chemical interactions exist between the polymer and the nanofillers [39]. Furthermore, it has been reported [40] that the thermal resistance at the interface between carbon nanomaterials and polymers decreases with the creation of chemical bonds, resulting in higher thermal conductivity that promotes heat dissipation within the sample. Thus, the increments found herein are significantly higher than those reported for PPy/rGO and PPy/graphene nanosheet nanocomposites prepared via in situ polymerization with nanofiller contents up to 20 and 40 wt%, respectively [41,42], which further corroborates the efficiency of the polymer-nanofiller covalent bonding to improve the thermal stability. A similar behavior of thermal stability improvement, with increments in T_max_ in the range of 40–65 °C, has been described for modified poly(ether ether ketone) (PEEK) derivatives covalently anchored to single-walled carbon nanotubes (SWCNTs) functionalized with carboxylic acids [16]. In this study, the superior increment observed for the sample prepared via conversion of the carboxylic groups to acyl chloride should be related to its greater number of covalent linkages. Residue data of the different samples at 600 °C are collected in Table S1. The values obtained for the grafted samples are consistent with their HDI-GO percentage taking into account that the HDI-GO loses about 48% of its weight below 600 °C, and that the polymer is not completely decomposed at such temperature.

### 3.4. XRD Analysis 

**Figure 6:**
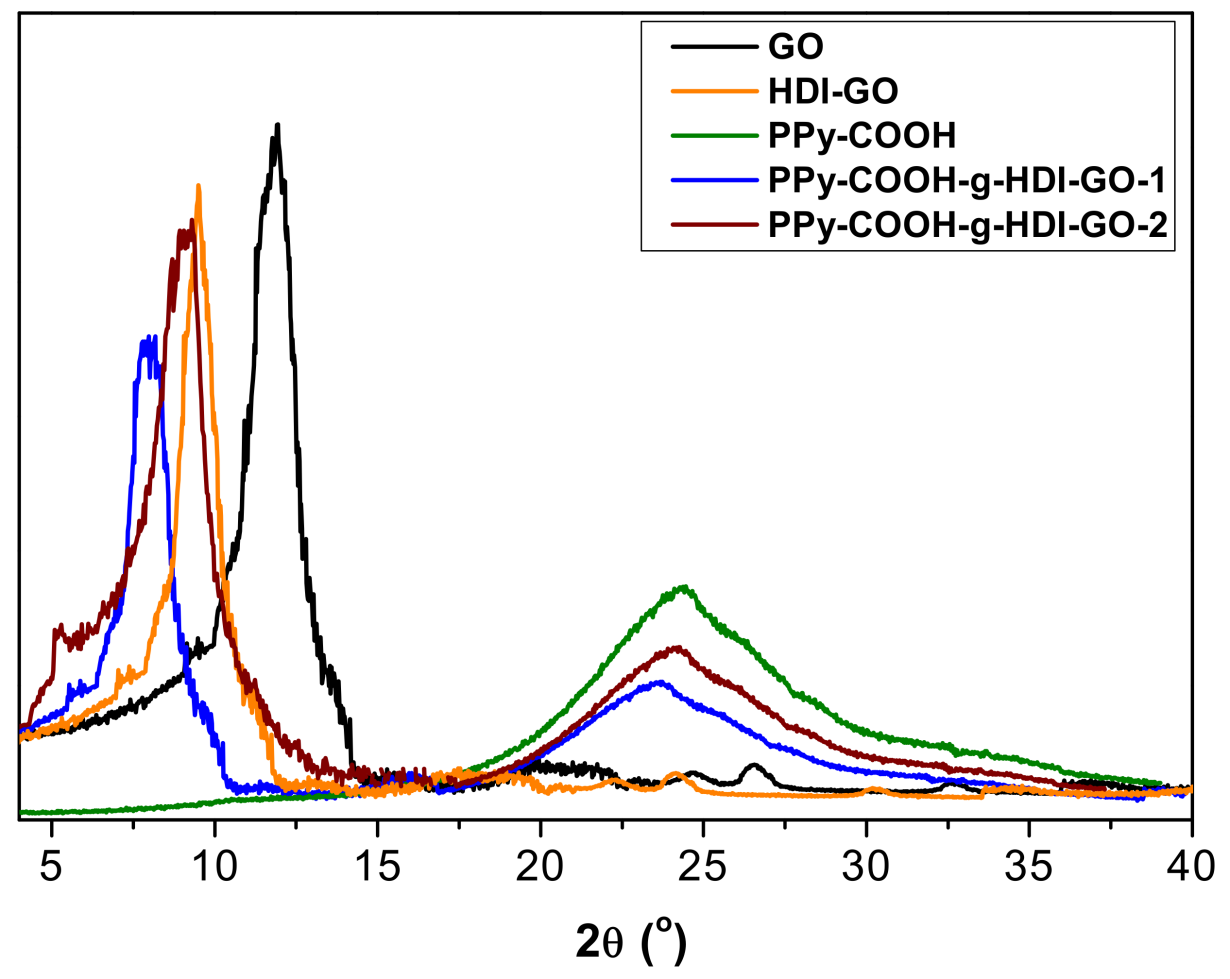
XRD patterns of PPy-COOH, GO, HDI-GO, and the grafted samples.

The different samples were also analyzed by XRD measurements, and representative diffractograms of PPy-COOH, GO, HDI-GO and the two grafted samples are compared in Figure 6. GO shows a typical peak at 2θ = 11.8° ascribed to the (002) crystalline plane [11], which corresponds to an interlayer *d* spacing of 0.748 nm according to the Bragg’s equation [43]. In the HDI-GO, the diffraction peak shows lower intensity and appears at 2θ = 9.2°, which corresponds to a *d* value of 0.961 nm. This increase in the *d* spacing is ascribed to the intercalation of the alkyl chains between the GO layers, as reported previously for other polymer/GO nanocomposites [44,45]. 

Regarding PPy-COOH, a broad peak is found in the region 17° < 2θ < 30°, revealing its amorphous nature. Such a wide peak indicates the absence of ordered arrangement of the chains, and is consistent with the structure reported in the literature [46]. This broad reflection is also found in the patterns of the grafted samples, although it appears at lower 2θ values, suggesting that the chains are more separated. Further, its intensity decreases, which is consistent with the reduction in the percentage of PPy-COOH upon HDI-GO grafting. These results are in contrast with those reported for PPy/graphene nanosheets, where the polymer grew along the nanomaterial surface during the in situ polymerization, resulting in a more-ordered structure [42]. On the other hand, a downshift of the (002) peak is also observed here in comparison with both GO and HDI-GO (Figure 6), corroborating the rise in the interlayer spacing of the carbon nanomaterial. The increment is higher for PPy-COOH-g-HDI-GO-1, in agreement with its larger degree of exfoliation and lower flake thickness (as indicated by TEM) and its higher SSA (calculated from BET measurements). Overall, XRD patterns confirm the effectiveness of the grafting process developed herein to aid the dispersion of the PPy-COOH chains within the interlayer spacing of the HDI-GO sheets.

### 3.5. Sheet Resistance 

The influence of HDI-GO on the electrical properties of PPy-COOH was investigated by measuring the sheet resistance (R_S_), and the results are collected in Table S1. Raw GO and HDI-GO were found to be electrically insulating, since the functional groups on the GO surface disrupted the conjugated π-electron system of the graphene sheets and hence it was not possible to measure their R_S_ value. In contrast, PPy-COOH displayed a low sheet resistance of about 180 Ω/sq, in agreement with the high electrical conductivity reported in previous studies [47]. This good electrical performance is attributed to the compact and smooth surface morphologies of the polymer nano- and meso-particles, as revealed by SEM analysis. As expected, the grafted samples show higher R_S_ than neat PPy-COOH, due to the anchoring of an insulating nanomaterial to the polymeric chains. Nonetheless, the values obtained (330 and 253 Ω/sq for PPy-COOH-g-HDI-GO-1 and PPy-COOH-HCI-GO-2, respectively) are lower than those estimated when considering the percentage of HDI-GO in the nanocomposites by a simple rule of mixtures. The discrepancy could be explained by taking into account the large number of factors that affect the conduction mechanism in this polymer, such as level of crystallinity, grain size, changes in the conformation of the polymeric chains, and doping and screening effects [48]. PPy-COOH possesses π electrons, since its structure contains alternate double and single bonds. Electrical conductivity in this polymer originates from the motion of electrons, or from positively charged carriers along the chains and the hopping of these carriers among chains [48]. Throughout the chemical polymerization, partial oxidation of the chains results in the formation of positive charges on the polymer backbone. The electrons of the π cloud of GO could settle near the positive charges to maintain neutrality of PPy backbone. Thus, the HDI-GO could act as a counterion of the oxidized form of PPy-COOH. This would result in the formation of PPy-COOH-HDI-GO charge-transfer complexes via strong donor–acceptor interactions, hence improving charge carrier transport. Further, the large SSA of HDI-GO could facilitate the charge transfer along the chains. All these facts could account for the relatively high electrical conductivity found for the grafted samples, despite the high electrical resistance of HDI-GO.

### 3.6. Mechanical Properties 

**Figure 7:**
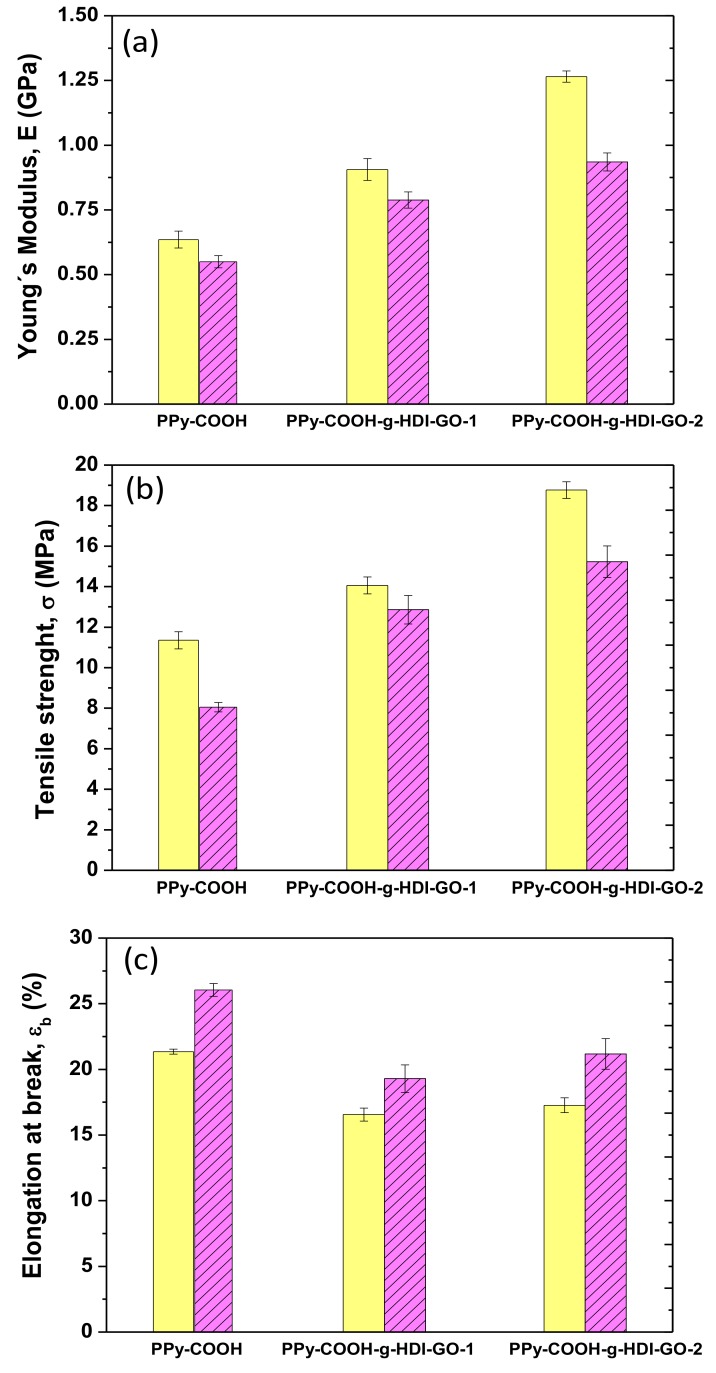
Tensile properties of PPy-COOH and the grafted samples at 25 °C (solid bars) and 100 °C (dashed bars): Young´s modulus (**a**), tensile strength (**b**) and elongation at break (**c**).

The mechanical properties of PPy-COOH and the grafted samples were explored by tensile measurements at 25 and 100 °C, and their Young´s (or elastic) modulus, tensile strength, and elongation (or strain) at break are plotted in Figure 7. At 25 °C, PPy-COOH exhibits elastic modulus and tensile strength values of 0.62 GPa and 11 MPa, respectively. The grafting of the HDI-GO causes large enhancements in both modulus and strength (Figure 7a,b) by about two-fold and 73%, respectively, for the sample with the highest grafting efficiency. An analogous tendency, though with somewhat lower increases, is found for the PPy-COOH-g-HDI-GO-1. The exceptional modulus and strength improvements attained demonstrate the high reinforcing efficiency of HDI-GO, likely arising from the strong PPy-COOH-HDI-GO interface adhesion achieved by the covalent bonding, therefore very effective load transfer from the PPy-COOH to the nanofiller sheets. A similar percentage of modulus increment was reported for a PEEK derivative covalently anchored to acid-functionalized SWCNTs [16]. The higher increment observed for PPy-COOH-g-HDI-GO-2 in comparison with the other grafted sample is likely associated with its higher yield of grafting. Furthermore, it is important to note that these samples contain a high GO content (~10 wt%), higher than the quantity typically incorporated in polymeric composites [49]. 

Focusing on the elongation at the break (Figure 7c), PPy-COOH presents a value close to 21% at 25 °C, which decreases upon the grafting of HDI-GO, the drop being ~20% for both grafted samples. This is the characteristic behavior reported for polymer/nanofiller composites [50], given that the fillers, especially those covalently bonded to the polymer, confine the ductile flow of the polymeric segments. Further, the strong adhesion between PPy-COOH and HDI-GO achieved by means of hydrogen bonding and electrostatic and π-π stacking interactions also promotes the decreased plasticity. Interestingly, the fall in ductility is similar for PPy-COOH-g-HDI-GO-1 and PPy-COOH-g-HDI-GO-2, despite the second exhibiting a higher grafting yield; hence PPy-COOH-g-HDI-GO-2 would be expected to show lower elongation at break. This could be due to the more-homogenous dispersion of the HDI-GO within the PPy-COOH polymer matrix, as suggested by SEM, since the presence of small GO agglomerates would limit the deformation of the matrix chains [22]. Thus, the grafting of carbon nanomaterials to polymer derivatives through in situ polymerization reactions leads to nanocomposites with more homogenously dispersed nanofillers than those prepared via direct integration using conventional methods such as melt blending or solution casting, and the higher the efficiency of the grafting reaction, the more homogeneous the resulting nanocomposites [51].

For certain applications, like photovoltaics [52] or high-temperature electronics, it is interesting to analyze the effect of temperature on the mechanical properties, stiffness in particular. Thus, the values of the modulus, strength, and ductility were also measured at 100° C, and the results are shown in Figure 7. Systematically, the stiffness and strength decrease with increasing temperature, due to the plasticization effect of the polymeric chains at high temperatures [53]. Nonetheless, the overall reinforcing efficiency of the HDI-GO seems to be comparable at both temperatures. Accordingly, the maximum improvements in stiffness and strength compared to PPy-COOH are about 80% and 90%, respectively, for the PPy-COOH-g-HDI-GO-1 sample. This confirms that the chain mobility constraint imposed by the grafted HDI-GO nanosheets is maintained at high temperatures. These results are in agreement with those described for polypropylene filled with similar loadings (~8.0 wt%) of inorganic nanoparticles, in which the reinforcing efficiency hardly changes with increasing temperature [54]. Conversely, a rise in the elongation at break is found with increasing temperature, which is the typical behavior expected for amorphous and semicrystalline polymeric materials. As the temperature increases, the amorphous regions become more mobile and this higher mobility is reflected in higher ductility. Compared to the neat polymer, a drop in ductility is again found for the grafted samples, the extent of the diminution being about 21% and 17% for PPy-COOH-g-HDI-GO-1 and PPy-COOH-g-HDI-GO-2, respectively. The drop in percentage is of the same magnitude to that found at 25 °C, being in this case slightly higher for the sample with the lower grafting yield, which is likely attributed to its less-homogenous HDI-GO dispersion, as discussed above. Overall, results confirm that mechanical properties that are dominated by the matrix, such as tensile strength, are strongly improved by the covalent grafting of a GO derivative, and that the reinforcing effect is preserved at high temperatures.

## 4. Conclusions

The covalent anchoring of PPy-COOH onto the surface of HDI-modified GO was performed by two different esterification reactions, and the grafting yields (about 31% and 42%) were estimated from TGA. Morphological observations via SEM and TEM showed an increase in the HDI-GO flake thickness for the sample synthesized via conversion of the carboxylic groups to acyl chloride, while it decreased slightly for that prepared by activation of the carboxylic acids by carbodiimide. FT-IR and Raman corroborated the grafting success, revealing the emergence of bands associated with ester linkages. According to XRD, the grafted samples displayed larger interlayer spacing than HDI-GO. They exhibited higher thermal stability than PPy-COOH, ~30 and 40 °C in the second degradation stage and improved stability was found for the sample with the greater number of covalent bonds. They also showed relatively low sheet resistance (330 and 253 Ω/sq, respectively), albeit higher than that of PPy-COOH (180 Ω/sq). Tensile tests demonstrated an exceptional enhancement in the Young´s modulus and tensile strength of PPy-COOH upon grafting the HDI-GO (by up to two-fold and 73%, respectively) for the sample synthesized via formation of the acyl chloride-functionalized PPy at 25 °C. Further, the reinforcing efficiency was approximately maintained at high temperatures. All the experiments performed prove that the use of a PPy derivative is a successful approach to anchor conductive polymers onto graphene-based nanomaterials for the manufacturing of nanocomposites to be used in flexible electronics, supercapacitors, solar cells, fuel cells, and so on. 

## Supplementary Materials

The following are available online at https://www.mdpi.com/2079-4991/9/8/1095/s1. Table S1: TGA, surface area and sheet resistance data of GO, HDI-GO, PPy-COOH, and the grafted samples.
