# Supplementary material for: Grafting of Polypyrrole-3-carboxylic Acid to the Surface of Hexamethylene Diisocyanate-Functionalized Graphene Oxide
Source: Nanomaterials (Basel). 2019 Jul 31;9(8):1095. doi: 10.3390/nano9081095 (PMC6723346; doi:10.3390/nano9081095)
Supplement: Supplementary file 1 [file nanomaterials-09-01095-s001.pdf]

Supplementary Materials

Grafting of polypyrrole-3-carboxylic acid to the surface of hexamethylene diisocyanate-functionalized graphene oxide

José Antonio Luceño-Sánchez[1] and Ana Maria Díez-Pascual[1,*]

[1] Department of Analytical Chemistry, Physical Chemistry and Chemical Engineering, Faculty of Sciences, Alcalá University, 28805 Madrid, Spain; jose.luceno@uah.es

* Correspondence: am.diez@uah.es; Tel.: +34-918-856-430

Table S1. TGA, surface area and sheet resistance data of GO, HDI-GO, PPy-COOH and the grafted samples.

Sample	T_i (ºC)	T_{10} (ºC)	$T_{max(I,II)}$ (ºC)	R (wt%)	SSA (m^2/g)	Rs (Ω/sq)
GO	121	186	222, -	48.5	103.1	-
HDI-GO	190	298	225, 387	48.2	81.2	-
PPy-COOH	202	257	276, 444	8.6	25.4	181
PPy-COOH-g-HDI-GO-1	204	255	271, 472	13.1	58.3	330
PPy-COOH-g-HDI-GO-2	195	271	290, 486	13.5	49.0	253

T_i: initial degradation temperature at 2% weight loss; T_{10}: temperature of 10% of weight loss. T_{max}: temperature of maximum rate of weight loss. The subscripts I and II refer to the first and second degradation stages, respectively. R: residue at 600 ºC. Rs: sheet resistance; SSA: specific surface area
